# Vertical Strata and Stem Carbon Dioxide Efflux in *Cycas* Trees

**DOI:** 10.3390/plants9020230

**Published:** 2020-02-11

**Authors:** Thomas E. Marler, Murukesan V. Krishnapillai

**Affiliations:** 1 College of Natural and Applied Sciences, University of Guam, Mangilao, Guam 96923, USA; 2 Cooperative Research and Extension, College of Micronesia-FSM, Yap Campus, Yap 96943, Micronesia; muru@comfsm.fm

**Keywords:** conservation physiology, *Cycas edentata*, *Cycas micronesica*, *Cycas nitida*, *Cycas revoluta*, *Cycas wadei*, *Cycas zambalensis*, stem respiration

## Abstract

Stem respiration is influenced by the vertical location of tree stems, but the influence of vertical location on stem respiration in a representative cycad species has not been determined. We quantified the influence of vertical strata on stem carbon dioxide efflux (*E*_s_) for six arborescent *Cycas* L. species to characterize this component of stem respiration and ecosystem carbon cycling. The influence of strata on *E*_s_ was remarkably consistent among the species, with a stable baseline flux characterizing the full mid-strata of the pachycaulous stems and an increase in *E*_s_ at the lowest and highest strata. The mid-strata flux ranged from 1.8 μmol·m^−2^·s^−1^ for *Cycas micronesica* K.D. Hill to 3.5 μmol·m^−2^·s^−1^ for *Cycas revoluta* Thunb. For all species, *E*_s_ increased about 30% at the lowest stratum and about 80% at the highest stratum. A significant quadratic model adequately described the *E*_s_ patterns for all six species. The increase of *E*_s_ at the lowest stratum was consistent with the influence of root-respired carbon dioxide entering the stem via sap flow, then contributing to *E*_s_ via radial conductance to the stem surface. The substantial increase in *E*_s_ at the highest stratum is likely a result of the growth and maintenance respiration of the massive cycad primary thickening meristem that constructs the unique pachycaulous cycad stem.

## 1. Introduction

Carbon dioxide is among the greenhouse gases that are emitted by tree stems and the amounts are great enough for this source of carbon dioxide to influence regional and global carbon cycles [1,2]. Vertical variations in stem respiration have been reported, with the stem surfaces directly above the root collar exhibiting greater carbon dioxide efflux (*E*_s_) than stem surfaces at higher strata [3,4,5]. Interpretations focus on respired carbon dioxide in root tissues that enters root xylem then moves to stem tissues in sap flow. This root-derived carbon dioxide responds to a radial conductance gradient toward the free air at these basal stem strata and some estimates indicate that about half of the root-respired carbon dioxide may enter the atmosphere by way of *E*_s_ [3]. The ability of stem xylem to dissolve locally respired carbon dioxide then transport it in the transpiration stream may also influence *E*_s_ in higher strata and is evinced in diel cycles of *E*_s_ as the changes in sap flow directly modify the conductance of carbon dioxide toward the stem surface [6,7,8,9]. As a result of sap flow and localized storage or refixation, about 40% of the respired carbon dioxide in stems may not be emitted from stem surfaces closest to the site of respiration [10].

A direct determination of the influence of vertical strata on tree *E*_s_ has been the subject of several studies [4,5,11,12,13,14,15,16]. Results either revealed little influence of vertical strata on *E*_s_ or indicated that an increase in *E*_s_ occurred in the highest strata of a tree’s bole and branches. The reported increase in *E*_s_ at higher strata may be a result of greater respiration in the more active stem tissues in the strata that are closest to the highly active leaf crown, a general reduction in the resistance to radial carbon dioxide conductance in the smaller branches at higher strata, and/or the influence of vertical strata on refixation or storage of carbon dioxide in stem tissues.

The literature on *E*_s_ is biased toward one stem form: pycnoxylic eudicot and gymnosperm trees that increase in stem diameter by bifacial secondary vascular cambium. Cycads produce manoxylic stems and, to our knowledge, only one report on *E*_s_ of cycads exists [17]. Cycads comprise an interesting group of gymnosperms that portray primitive features and cycads are intriguing to botanists because of their antiquity, unique traits, and beauty [18]. The cycad stem is constructed in a unique manner to produce a pachycaulous form that contains persistent pith and cortex and is comprised mostly of living parenchyma tissues [18,19]. Radial enlargement of the cycad stem is mostly restricted to the primary thickening meristem [20,21,22], the largest known apical meristem in the plant kingdom [18]. Clearly, our current level of knowledge of *E*_s_ is deficient because manoxylic, pachycaulous trees have been ignored in the research to date.

The simplicity of cycad stem architecture removes some of the complications in interpreting the influence of tree height on *E*_s_. Indeed, the published studies on the subject reported *E*_s_ from the bole for lowest strata but from small side branches for the highest strata of the canopy. Stem diameter and radial organization of tissues may exert a direct influence on *E*_s_ [11,14], so the influences of vertical strata on *E*_s_ of these species may be mediated via the canopy architecture and stem design rather than via vertical strata per se. The pachycaulous cycad stem is essentially a cylinder of similar diameter for most of the tree height, providing a model tree form where the vertical gradient of *E*_s_ can be studied without the complication of elaborate branch architecture and disparate internal organization of different sized stems.

**Figure 1:**
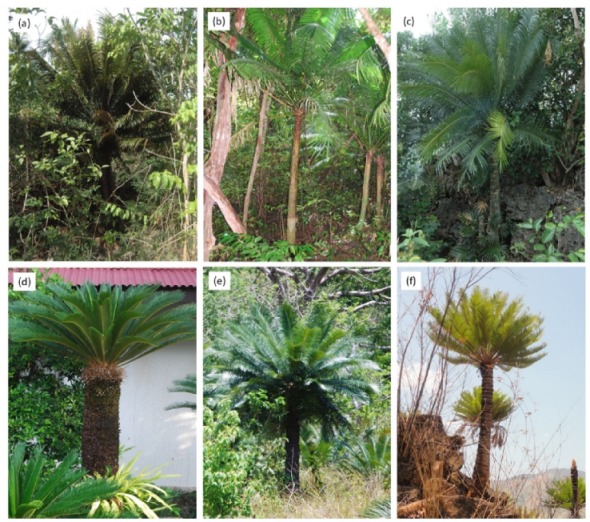
The general appearance of six *Cycas* tree species in the study locations. (**a**) *Cycas edentata* in an agroforest in Angeles City, Philippines; (**b**) *Cycas micronesica* in native Yap habitat; (**c**) *Cycas nitida* in its native northern Samar, Philippines habitat; (**d**) *Cycas revoluta* in an urban landscape in Angeles City, Philippines; (**e**) *Cycas wadei* in its endemic Culion Island, Philippines habitat; (**f**) *Cycas zambalensis* in its endemic San Antonio, Philippines habitat.

Our objectives were to determine the influence of vertical strata on *E*_s_ for six representative arborescent *Cycas* species (Figure 1). We predicted little influence of vertical strata on *E*_s_ from the root collar to the mid-strata of each tree, then an increase in *E*_s_ close to the stem apex due to respiratory activity of the highly-active primary thickening meristem.

## 2. Results

**Figure 2:**
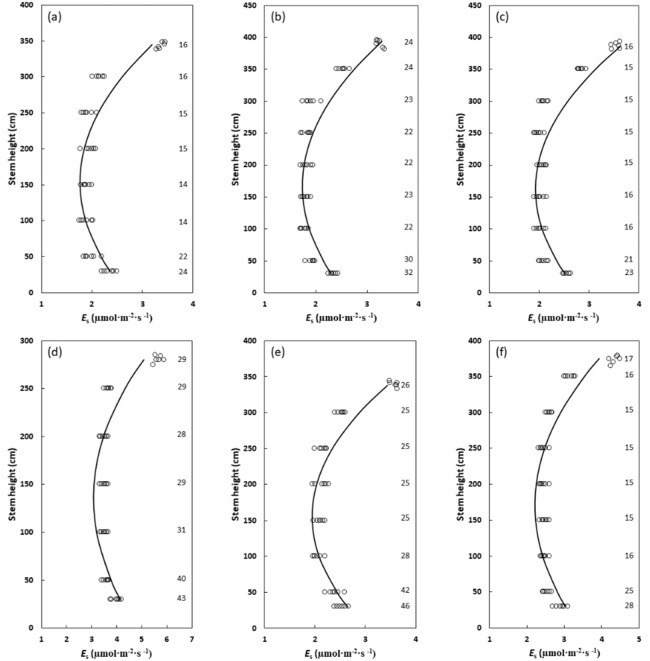
The relationship between vertical strata and stem carbon dioxide efflux (*E*_s_) for six arborescent *Cycas* species. (**a**) *Cycas edentata*; (**b**) *Cycas micronesica*; (**c**) *Cycas nitida*; (**d**) *Cycas revoluta*; (**e**) *Cycas wadei*; and (**f**) *Cycas zambalensis*. Solid lines represent the quadratic model calculated from the mean of each parameter after fitting the data for each of the six replications per species, with axes transposed. Numbers on the right vertical axis represent the mean stem diameter for the six replications of each species. Models for each species were: (**a**) *E*_s_ = 2.63 − 0.01*height + 0.00004*height^2^; *P* < 0.001; *R^2^* = 0.78; (**b**) *E*_s_ = 2.54 − 0.01*height + 0.00003*height^2^; *P* < 0.001; *R^2^* = 0.88; (**c**) *E*_s_ = 2.77 − 0.01*height + 0.00003*height^2^; *P* < 0.001; *R^2^* = 0.87; (**d**) *E*_s_ = 4.61 − 0.22*height + 0.00008*height^2^; *P* < 0.001; *R^2^* = 0.71; (**e**) *E*_s_ = 2.93 − 0.01*height + 0.00004*height^2^; *P* < 0.001; *R^2^* = 0.85; (**f**) *E*_s_ = 3.24 − 0.01*height + 0.00004*height^2^; *P* < 0.001; *R^2^* = 0.79.

Midday air temperature, stem surface temperature, and relative humidity were similar among the six locations of study. The tallest stratum of measurements ranged from 2.8 m for *C. revoluta* to 3.9 m for *C. micronesica* and *C. nitida* (Figure 2). The mid-section of the stems exhibited homogeneous *E*_s_ for all six arborescent *Cycas* species in this study (Figure 2). In ascending order, the mid-strata *E*_s_ was 1.8 μmol·m^−2^·s^−1^ for *C. micronesica*, 1.9 μmol·m^−2^·s^−1^ for *Cycas edentata* de Laub., 2.0 μmol·m^−2^·s^−1^ for *Cycas nitida* K.D. Hill & A. Lindstrom, 2.1 μmol·m^−2^·s^−1^ for *Cycas wadei* Merrill, 2.5 μmol·m^−2^·s^−1^ for *Cycas zambalensis* Madulid & Agoo, and 3.5 μmol·m^−2^·s^−1^ for *C. revoluta*.

The *E*_s_ of the lowest stratum for all six species increased about 30% above that of the mid-strata (Figure 2). An increase in stem diameter also occurred at the lowest strata of the stems (Figure 2). The *E*_s_ at the lowest stratum was 2.4 μmol·m^−2^·s^−1^ for *C. micronesica* and *C. edentata*, 2.6 μmol·m^−2^·s^−1^ for *C. nitida* and *C. wadei*, 3.1 μmol·m^−2^·s^−1^ for *C. zambalensis*, and 4.1 μmol·m^−2^·s^−1^ for *C. revoluta*. An 80% increase in *E*_s_ above the mid-strata *E*_s_ occurred in the highest measurement stratum (Figure 2). This apical stem *E*_s_ was 3.3 μmol·m^−2^·s^−1^ for *C. micronesica*, 3.4 μmol·m^−2^·s^−1^ for *C. edentata*, 3.6 μmol·m^−2^·s^−1^ for *C. nitida* and *C. wadei*, 4.5 μmol·m^−2^·s^−1^ for *C. zambalensis*, and 5.9 μmol·m^−2^·s^−1^ for *C. revoluta*.

The influence of vertical strata on *E*_s_ could be fitted with significant quadratic regression models for every individual and every species (Table 1, Figure 2). The *E*_s_ of the second lowest stratum was more similar to the mid-strata *E*_s_ than the *E*_s_ at the lowest stratum for these *Cycas* trees. In contrast, the *E*_s_ of the second highest stratum revealed a moderate increase above that of the mid-strata *E*_s_ for four of the *Cycas* species, but not for *C. edentata* or *C. revoluta*.

**Figure 3:**
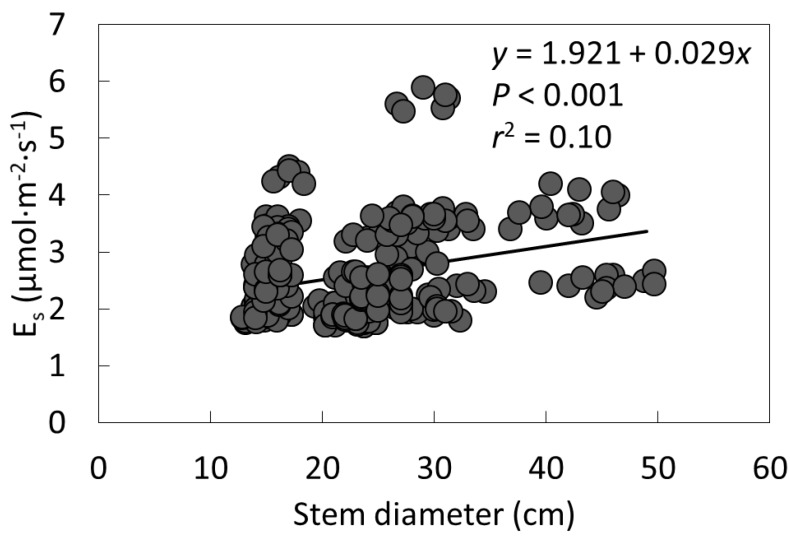
The relationship between stem diameter and stem carbon dioxide efflux (*E*_s_) for six arborescent *Cycas* species.

Stem carbon dioxide efflux increased with stem diameter (Figure 3). The inclusion of 300 data pairs in the regression generated a highly significant linear model, but the considerable scatter reduced the fitness.

## 3. Discussion

### 3.1. Cycad Stems

The homogeneous cycad pachycaulous stem construction design [23] may explain the similarity in *E*_s_ for our mid-strata measurements. Pachycaulous stem forms have evolved in other plant groups, such as palms and other arborescent monocot species, but the cycad stem is constructed by the largest known primary thickening meristem of any plant group [18]. Radial enlargement in sections of the cycad stem subtending the leaf crown occurs by way of lethargic disjunct mitotic activity that is scattered among the tissues throughout the cross-section of the stem [24]. These traits of the cycad pachycaulous stem may explain the remarkable similarity in *E*_s_ throughout most of the vertical strata of the *Cycas* stems for all six species that we studied and this partly conformed to our first prediction.

The moderate increase in *E*_s_ at the lowest stratum was not consistent with our first prediction. Four anatomical and physiological features of the cycad stem may explain this increase in *E*_s_ near the root collar. First, increased *E*_s_ at basal stem strata may result from the contribution of root-respired carbon dioxide that is xylem-transported to the stem [3,4,5]. Second, the peripheral tissues of a cycad stem are comprised of a distinct corky periderm layer and persistent leaf petiole scars that include living and dead structures [18,23,25]. The thickness of this cork tissue is greatest at the base of cycad trees [18]. The secondary vascular cambium and bark tissues of pycnoxylic eudicot and gymnosperm trees may act as conductance barriers to gases [26,27]. Similarly, the peripheral structures of a cycad stem may act as conductance barriers in a manner that influences carbon dioxide diffusion to the open air and this trait may vary among cycad species and along the vertical axis of the pachycaulous cycad stem. Third, the absolute diameter of the cortex increases with cycad stem height and cortex width may influence *E*_s_ [18,24]. Fourth, vascular strands traverse the cycad stem cortex in a non-linear manner, connecting the vascular cylinders with each leaf, and these strands persist in the cortex tissue following leaf mortality [18,22,28,29]. These vascular strands at the base of a tall cycad tree may be decades or centuries in age, yet the vascular strands near the stem apex are relatively young. The need to more fully understand these vertical gradients in tree *E*_s_ in the face of ongoing climate change is underscored by the reported influence of elevated atmosphere carbon dioxide concentration on the relationship between vertical strata and *E*_s_ [30].

The substantial increase in *E*_s_ at the highest stem stratum conformed to our second prediction. The mitotic tissues that construct radial cycad stem growth are uniquely constricted to the massive primary thickening meristem [20,21,22], so restriction of the greatest growth and maintenance respiration to the stem stratum that contains this structure was expected. The relative *E*_s_ of the second highest stem stratum varied among the six species. This contrast in behavior was probably a function of how close these measurements were to the primary thickening meristem in each replication’s stem apex, which was controlled by the axial location of the oldest living petioles of the leaf crowns of each species. The poorer fit of the quadratic models for *C. edentata* and *C. revoluta* than for the other species (Table 1) was likely due to the fact that the observed *E*_s_ values of this second highest stratum for these two species were less aligned with the non-linear upper portions of the regressions’ predicted values.

The range in *E*_s_ was 1.9-fold for the baseline flux of the mid-sections of stems for these *Cycas* species, the range in *E*_s_ was 1.8-fold for the flux near the leaf crown, and the range in *E*_s_ was 1.7-fold for the flux near the root collar. Therefore, the variation of *E*_s_ among *Cycas* plants appears more constrained at basal and apical stem locations than at mid-locations of the stem. This variation among only six congeneric cycad taxa reveals the need to add more pachycaulous tree species to the global *E*_s_ database.

### 3.2. Stem Respiration Literature Bias

The *E*_s_ literature is comprised almost exclusively of pycnoxylic eudicot and gymnosperm tree species that grow radially by bifacial secondary vascular cambium. We are aware of only one other report that included *E*_s_ of pachycaulous tree species. An impressive data set collection of woody tissue carbon dioxide efflux was conducted in Costa Rica and one growth form in this study was palms [14]. The arborescent palm stem is also classified as pachycaulous, however, the palm stem anatomy and methods of radial stem growth are inconsistent with cycad stems. One similarity is evident from the Costa Rica palm results and our cycad results: the mid-strata of the pachycaulous palm and cycad stem is essentially a cylinder with minimal variation in *E*_s_. The *E*_s_ of this mid-section was less than 1 μmol·m^−2^·s^−1^ for the palm stems (no species were named) in the Costa Rica study [14] and up to 3.5 μmol·m^−2^·s^−1^ for the *Cycas* stems in our study.

Our understanding of *E*_s_ is hindered by the lack of data on tree species with growth forms that do not expand radially by secondary vascular cambium. We have addressed this limitation by adding six cycad species to the literature. The generality of the quadratic pattern of the relationship between stem height increment and *E*_s_ remains unexplored for other arborescent cycad taxa. Clearly, more data are urgently needed for cycads, palms, and other monocot arborescent species to fully understand *E*_s_ across all tree species and develop models that adequately predict all arborescent functional groups. Ecosystem carbon cycling models based on the current *E*_s_ literature are not relevant for localities with numerous palms, cycads, or other monocot trees.

## 4. Materials and Methods

Access to a large number of tall cycad trees within homogeneous edaphic and climatic conditions is difficult to achieve. We addressed these limitations by accessing high-density in situ localities for four *Cycas* species and urban landscape specimens within one municipality for two common garden species. For each species, the populations were carefully surveyed for the tallest available, containing at least six representative trees. Although cycads do not produce axillary buds during primary growth, the stems do possess the ability to produce adventitious buds that generate branching [18]. We restricted the selection of replications to monopodial individuals and did not include trees with multiple branches. The initiation of reproductive structures on dioecious cycad trees is infrequent and we did not include any trees with developing reproductive structures. These methods ensured that this first look at cycad *E*_s_ was not complicated by ambiguities introduced by multi-stem trees and sink activity of expanding reproductive organs, which are always positioned at the stem apex. These methods also excluded the use of the tallest individuals in each locality in order to enable appropriate replication.

**Figure 4:**
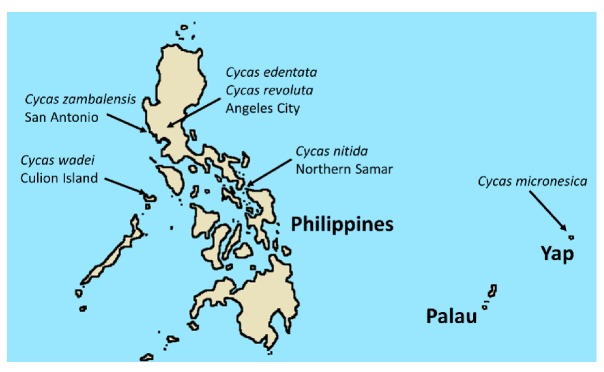
Map of the study locations in western Pacific islands.

In situ *Cycas micronesica* trees were studied from 5 to 10 Feb 2018 on Yap island (Figure 4). The locality was a closed-canopy forest growing on acid schist soils that have been the subject of several investigations [31,32,33,34]. Stem heights of the replications were 405–415 cm. Slope, as determined by a clinometer, was 21° to 32°. In situ *Cycas nitida* trees were studied from 10 to 14 Mar 2019 in a habitat located on a small barrier island in Northwestern Samar near the San Bernardino Strait. The population was growing 20–30 m from the shore as understory components of littoral forests in sand substrate with karst outcrops and had been the subject of one investigation [35]. Stem heights of the replications were 415–425 cm and slope was 2° to 4°. In situ *Cycas zambalensis* trees were studied on 27 Apr 2019 in San Antonio, Zambales, Philippines. The population was growing in full sun conditions in ultramafic soils. Stem heights of the replications were 405–415 cm and slope was 34° to 46°. In situ *Cycas wadei* trees were studied on 4 May 2019 within the only known endemic population on Culion Island, Philippines. The plants studied were growing in full sun conditions in impoverished soils and the locality has been the subject of one investigation [36]. Stem heights of the replications were 355–365 cm and slope was 5° to 17°.

*Cycas edentata* and *Cycas revoluta* garden trees in the Angeles City, Philippines urban landscape were studied from 16 to 24 Dec 2019. These two cycad species have long been favored specimens for municipal landscapes in the Philippines, where they are used at City Hall, Barangay Hall, and school buildings. Old trees with substantial heights are easily located in most municipalities. The native soil is a neutral coarse loam, but the landscape substrates had been heavily modified. The *C. edentata* stem heights were 355–370 cm and the *C. revoluta* stem heights were 300–315 cm. Angeles City is positioned on a plain, so slope was not measurable for any of the replications.

Stem vertical strata categories for *E*_s_ measurements were determined in a fixed manner for all six replications of all six species. The lowest stratum was fixed at 30 cm height, then vertical increments of 50 cm were included, beginning at a height of 50 cm, and continued until the ultimate height of each tree. The maximum stratum for stem measurements was adjusted to the position directly below the living petioles of the leaf crown for each tree. This approach generated fixed vertical strata for every height category except the tallest height category, which varied among the replications and species.

**Figure 5:**
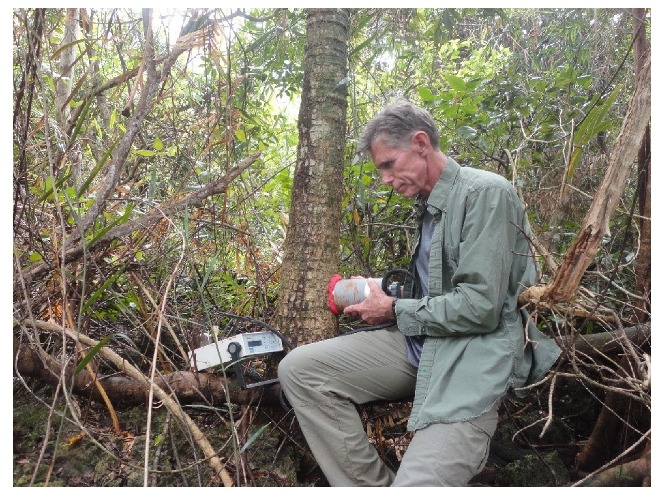
Depiction of the infrared gas exchange instrument (left) and chamber in use near the base of a *Cycas* stem with red malleable modeling clay used for seal.

A CIRAS EGM-4 analyzer fitted with a SRC-1 closed system chamber (PP Systems, Amesbury, MA, USA) was used to quantify the efflux of carbon dioxide from stem surfaces at each pre-defined stratum. The use of a horizontally-oriented soil chamber has been previously reported for tree *E*_s_ measurements [17,37,38]. A ring of modeling clay, approximately 10 cm in diameter, was placed on the stem surface to form a malleable seal, then the SRC-1 chamber was inserted into the modeling clay to provide a sealed chamber of 1.171 L volume (Figure 5). The use of modeling clay to form sealed gas exchange chambers on tree stems has been previously reported [17,39].

The EGM-4 recorded air temperature and the increase in carbon dioxide above ambient for a 2 min period. The change in carbon dioxide was used to calculate *E*_s_. Three periods of efflux were conducted for each measurement and the mean *E*_s_ was used as the value for each stem height for each replication. The stem surface temperature was measured with an infrared thermometer (Milwaukee Model 2267-20, Milwaukee Tool, Brookfield, WI, USA). Relative humidity was determined with a sling psychrometer. Stem diameter at the height of each flux measurement and total stem height were recorded. The time of day was limited to 10:00–14:00 to restrict time of day to the middle of the photoperiod as the diel cycle of *E*_s_ is not known for cycads.

The *E*_s_ data for each replication were plotted in scatter plots to reveal the general relationship of *E*_s_ to vertical strata. These plots revealed the approximate fit of a quadratic relationship, so the general linear model (Proc GLM, SAS Institute, Cary, NC, USA) was employed to fit the quadratic models for each replication. Then, one regression was conducted for each species to determine one universal quadratic model. We also used the Proc GLM to determine the linear relationship between stem diameter and *E*_s_.

## Figures and Tables

**Table 1:** Characteristics of the six study locations used for *Cycas* stem carbon dioxide efflux measurements. Range of six replications.

Species	Air Temperature (°C)	Stem Temperature (°C)	Relative Humidity (%)	Quadratic Model *P* Value	Quadratic Model R^2^
*Cycas edentata*	27–32	26–32	59–65	0.0079–0.0018	0.78–0.82
*Cycas micronesica*	28–31	26–31	60–69	0.0021–0.0011	0.88–0.90
*Cycas nitida*	26–30	25–30	63–71	0.0014–0.0018	0.87–0.90
*Cycas revoluta*	27–33	27–31	59–66	0.0087–0.0015	0.71–0.77
*Cycas wadei*	29–34	27–33	60–68	0.0500–0.0501	0.85–0.88
*Cycas zambalensis*	26–30	25–29	58–64	0.0139–0.0110	0.79–0.84
